# The prevalence and predictors of geriatric giants in community-dwelling older adults: a cross-sectional study from the Middle East

**DOI:** 10.1038/s41598-023-39614-4

**Published:** 2023-07-31

**Authors:** Seyedeh Zahra Badrkhahan, Moein Ala, Hossein Fakhrzadeh, Arash Yaghoobi, Sara Mirzamohamadi, Seyed Masoud Arzaghi, Sina Shahabi, Farshad Sharifi, Afshin Ostovar, Noushin Fahimfar, Iraj Nabipour, Bagher Larijani, Gita Shafiee, Ramin Heshmat

**Affiliations:** 1 Department of Geriatric Medicine, Tehran University of Medical Sciences (TUMS), Tehran, Iran; 2 Cardiac Primary Prevention Research Center, Cardiovascular Disease Research Institute, Tehran Heart Center (THC), Tehran University of Medical Sciences (TUMS), Tehran, Iran; 3 Experimental Medicine Research Center, Tehran University of Medical Sciences (TUMS), Tehran, Iran; 4 Elderly Health Research Center, Endocrinology and Metabolism Population Sciences Institute, Tehran University of Medical Sciences, Tehran, Iran; 5 School of Medicine, Tehran University of Medical Sciences (TUMS), Tehran, Iran; 6 Non-Commutable Disease Research Center, Endocrinology and Metabolism Population Sciences Institute, Tehran University of Medical Sciences (TUMS), Tehran, Iran; 7 Osteoporosis Research Center, Endocrinology and Metabolism Clinical Sciences Institute, Tehran University of Medical Sciences (TUMS), Tehran, Iran; 8 The Persian Gulf Marine, Biotechnology Research Center, The Persian Biomedical Sciences Research Institute, Bushehr University of Medical Sciences, Bushehr, Iran; 9 Endocrinology and Metabolism Research Center, Endocrinology and Metabolism Clinical Sciences Institute, Tehran University of Medical Sciences (TUMS), Tehran, Iran; 10 Chronic Disease Research Center, Endocrinology and Metabolism Population Sciences Institute, Tehran University of Medical Sciences (TUMS), Tehran, Iran

**Keywords:** Risk factors, Disease prevention, Geriatrics, Patient education, Quality of life, Disability

## Abstract

The term “geriatric giants” refers to the chronic disabilities of senescence leading to adverse health outcomes. This study aimed to investigate the prevalence and predictors of geriatric giants in Southern Iran. The participants were selected from Bushehr city using a multistage cluster random sampling method. Demographic data were collected through interviews. Frailty, incontinence, immobility, depression, cognitive impairment, and malnutrition were measured by questionnaires and instruments. Finally, data from 2392 participants were analyzed. The prevalence of fecal incontinence was less than 1% among all participants and similar in men and women. In contrast, compared with men, women had higher prevalence of urinary incontinence (36.44% vs. 17.65%), depression (39.05% vs. 12.89%), anorexia and malnutrition (2.35% vs. 0.82%), immobility (8.00% vs. 2.5%), frailty (16.84 vs. 7.34), and pre-frailty (54.19 vs. 38.63%). The prevalence of dependence and cognitive impairment was also higher in women and considerably increased with the age of participants. In total, 12.07% of subjects were frail, and 46.76% were pre-frail. The prevalence of frailty exponentially increased in older age, ranging from 4.18% among those aged 60–64 years to 57.35% in those aged ≥ 80 years. Considering 95% confidence interval (CI), multivariate logistic regression revealed that low physical activity [odds ratio (OR) 31.73 (18.44–54.60)], cancer (OR 3.28 (1.27–8.44)), depression [OR 2.42 (1.97–2.98)], age [OR 1.11 (1.08–1.14)], waist circumference [OR 1.03 (1.01–1.06)], BMI [OR 1.07 (1.01–1.14)], MNA score [OR 0.85 (0.79–0.92)], polypharmacy [OR 2.26 (1.30–3.95)] and male gender [OR 0.63 (0.42–0.93)] were independently associated with frailty. White blood cell count (WBC), smoking, marital status, and number of comorbidities were not independently associated with frailty. Low physical activity was the strongest predictor of frailty, which may need more attention in geriatric care. Frailty, its predictors, and other components of geriatric giants were considerably more common among women and older ages.

## Introduction

Life expectancy has markedly increased over the past decades mainly due to reduced mortality during early and middle life in the first half of the twentieth century and improved survival after 65 years of age in the second half of the twentieth century^1,2^. The prevalence of major chronic diseases generally increases with age and aging is accompanied by higher odds of several health conditions, particularly geriatric giants^3,4^. In 1965, Bernard Isaacs defined geriatric giants as “immobility, instability, incontinence, and impaired intellect/memory”. The definition was then modified by experts in the field. Different components of geriatric giants can interact with each other and one component can contribute to the incidence of the other known as the geriatric cascade, which propels old subjects from robustness to disability^5^. As they are assumed the natural consequence of senescence by patients, their caregivers, and even physicians, the giants of geriatrics often do not receive enough attention until they impose a significant burden on patients and the healthcare system^5,6^. Although there is not a unanimous definition, we considered frailty, immobility, instability, incontinence, cognitive impairment, anorexia, functional status, depression, and polypharmacy as geriatric giants in this study.

A recent report from Turkey with 2816 participants indicated that the prevalence of polypharmacy, urinary incontinence, depression, dementia, falls, sarcopenia, frailty, and malnutrition was as high as 54.5%, 47.6%, 35.1%, 21.6%, 33.6%, 31.7%, 28.3%, and 9.6%, respectively^7^. Another study from Turkey indicated that 90.3% of patients with Lewy body dementia and 54.9% of patients with Alzheimer's disease had ≥ 3 components of geriatric giants, reminding the effect of one component on the presence of other components^8^. Moreover, the presence of a pre-existing condition such as anemia was shown to increase the odds of geriatric giants^9^.

Frailty is the result of deficit accumulation in several body systems, which itself increases vulnerability to external stressors^10^. Not all old patients, but many of them, particularly those visiting health centers, experience frailty. Based on the definition of frailty and the characteristics of the target population, the prevalence of frailty and pre-frailty in most studies widely varies across different studies mainly from 5.9 to 17.4% and from 26.8 to 62.8%, respectively^11^. However, a study from the north of Iran with 2010 participants reported that the prevalence of frailty and pre-frailty was 33.4% and 43.5%, respectively^12^. In addition, a meta-analysis of 9 studies from the Middle East reported that the prevalence of pre-frailty and frailty was as high as 39% and 35%, respectively^13^. A study from France has estimated that pre-frailty and frailty caused 750 € and a 1500 € increment in ambulatory health expenditures for each individual just in 2012^14^. Clinical identification of frailty is necessary for promoting the adaptation of health care services to the needs. Recently, the prevalence of frailty has become a major index for evaluating the health status of the elderly population. On the other hand, healthcare policies have been modified to allocate more resources for preventing frailty or educating and rehabilitating those with frailty^15,16^. Herein, an interdisciplinary effort has been made in recent years to recognize frailty as a medical condition that needs early screening, advanced knowledge, and sufficient management^10,16^.

Having a thorough knowledge of the extent of the problem and its predictors is the main prerequisite for policy-making. Herein, in this community-based study, we measured the prevalence of geriatric giants and frailty in Southern Iran and simultaneously identified the associated factors. Identification of the prevalence of geriatric giants and frailty in Iran can better reflect the current status of geriatric health in this region. Particularly, a deeper insight into the associated factors of geriatric giants and frailty may help design better preventive and screening plans to promote geriatric health. Using a multistage cluster random sampling method, we included participants from the second wave of the Bushehr Elderly Health Program (BEHP)^17^.

## Results

Data were obtained from 2392 participants. Among them, 51.37% were female. The mean age of the participants was 70.13 years. The prevalence of obesity was 23.39% (95% CI 23.70–27.16) (Table 1).

**Table 1:** Characteristics of the participants.

Variables		All the participants (%) N = 2392	Non-frail (%) N = 1173	Pre-frail (%) N = 1026	Frail (%) N = 193
		Point estimation (95% CI)	Point estimation (95% CI)	Point estimation (95% CI)	Point estimation (95% CI)
Age (year)*		70.13 (70.07–70.19)	69.97 (69.84–70.10)	70.12 (70.03–70.21)	70.48 (70.31–70.65)
Gender (female)		51.37 (49.70–53.04)	33.61 (30.44–36.94)	60.25 (57.32–63.11)	74.99 (68.16–80.78)
Education	Illiterate	36.81 (34.93–38.98)	39.14 (35.41–42.99)	35.55 (32.55–32.57)	43.89 (36.18–51.90)
	Primary	35.09 (32.99–37.26)	33.67 (30.03–37.52)	35.39 (32.33–38.57)	33.17 (26.10–41.10)
	Secondary	8.66 (7.55–9.92)	7.11 (5.58–9.03)	9.14 (7.53–11.06)	11.10 (6.91–17.35)
	Diploma	12.80 (11.45–14.27)	14.17 (11.54–17.28)	12.88 (10.96–15.08)	8.41 (5.05–13.68)
	Academic	6.64 (5.65–7.78)	5.91 (4.57–7.61)	7.04 (5.58–8.84)	3.43 (1.48–7.73)
Marital status	Single	0.68 (0.42–1.09)	0.63 (0.31–1.28)	0.72 (0.35–1.49)	0.54 (0.08–3.69)
	Married	75.59 (73.62–77.47)	84.38 (81.01–87.25)	74.12 (71.21–76.82)	58.13 (49.75–66.06)
	Divorce	0.86 (0.55–1.34)	1.38 (0.63–2.98)	0.62 (0.30–1.30)	0.46 (0.12–1.80)
	Widow	22.87 (21.03–24.82)	13.61 (10.88–16.89)	24.54 (21.86–27.43)	40.87 (33.02–49.21)
BMI categorized	Underweight*	2.64 (2.04–3.42)	0.24 (0.07–0.83)	4.38 (3.23–5.88)	4.64 (2.08–10.00)
	Ideal weight*	29.62 (27.80–31.51)	27.53 (24.10–31.24)	30.06 (27.24–33.06)	34.05 (27.68–41.05)
	Overweight*	42.34 (40.42–44.28)	49.95 (46.26–53.64)	35.80 (32.79–38.92)	35.32 (28.22–43.13)
	Obese*	25.39 (23.70–27.16)	22.28 (19.22–25.67)	29.77 (26.96–32.74)	25.98 (19.65–33.51)

*WHO population age-standardized.

The WHO age-standardized prevalence of frailty was 12.07% (95% CI 10.89–13.36%) and the prevalence of pre-frailty was 46.76% (95% CI 44.72–48.81%) based on Fried frailty phenotype. The WHO age-standardized prevalence of frailty was 14.20% (95% CI 12.83–15.70%) and the prevalence of pre-frailty was 64.75% (95% CI 62.76–66.69%) based on frailty index. The prevalence of frailty increased with age. For instance, based on Fried frailty phenotype, the prevalence of frailty was 4.18% among those with 60–64 years of age and reached 57.35% among those ≥ 85 years of age. Based on frailty index, the prevalence of frailty was 7.59% among those with 60–64 years of age and reached 55.17% among those ≥ 85 years of age. Furthermore, the prevalence of frailty in females was more than twice of that in males based on the Fried frailty phenotype (16.84% vs. 7.34%, respectively) (Table 2). The prevalence of frailty in females was more than three times of that in males based on the frailty index (21.09% vs. 6.88%, respectively). The prevalence of frailty and pre-frailty in different age and sex groups based on frailty index has been summarized in Supp Table 1. The most common characteristic of all frail participants was low walking speed (34.16%), and the least common feature was weight loss (6.15%) (Supp Table 2).

**Table 2:** The prevalence of frailty based on Fried frailty phenotype according of age groups and genders.

Variables	Total Participants (%) N = 2392		Female (%) N = 1235		Male (%) N = 1157	
	Pre-frailty N = 1026	Frailty N = 193	Pre-frailty N = 602	Frailty N = 119	Pre-frailty N = 424	Frailty N = 74
Age groups	Point estimation (95% CI)	Point estimation (95% CI)	Point estimation (95% CI)	Point estimation (95% CI)	Point estimation (95% CI)	Point estimation (95% CI)
60–64 years	37.29 (33.60–41.13)	4.18 (2.83–6.13)	47.13 (41.90–52.43)	6.37 (4.17–9.61)	26.41 (21.55–31.92)	1.76 (0.74–4.13)
65–69 years	48.10 (44.92–51.30)	4.84 (3.57–6.54)	58.40 (54.20–62.48)	6.30 (4.48–8.77)	35.45 (30.93–40.24)	3.05 (1.65–5.58)
70–74 years	52.38 (47.35–57.36)	10.58 (7.92–13.99)	61.80 (54.45–62.48)	18.54 (13.63–24.71)	44.00 (37.48–50.73)	3.50 (1.69–7.10)
75–79 years	57.09 (51.64–62.38)	19.50 (15.44–24.32)	59.85 (51.95–67.26)	28.79 (22.12–36.52)	54.66 (47.15–61.97)	11.33 (7.27–17.24)
80–84 years	60.27 (52.02–67.98)	27.40 (20.89–35.03)	56.52 (44.15–68.13)	36.23 (25.89–48.03)	63.64 (52.23–73.69)	19.48 (12.04–29.95)
≥ 85 years	32.35 (22.27–44.39)	57.35 (45.56–68.36)	32.50 (20.10–47.95)	65.00 (49.53–77.85)	32.14 (17.32–51.71)	46.43 (29.72–63.98)
All age groups*	46.04 (43.95–48.15)	12.36 (11.15–13.68)	53.59 (50.77–56.39)	16.73 (14.86–18.79)	38.49 (35.50–41.58)	7.98 (6.41–9.90)
All age groups**	46.76 (44.72–48.81)	12.07 (10.89–13.36)	54.19 (51.39–56.95)	16.84 (14.95–18.91)	38.63 (35.75–41.59)	7.34 (5.91–9.07)

*Age and sex standardized based on the Iranian population in 2016.

**Age-standardized based on World Health Organization Population 2000–2025.

Using the χ^2^ test, we measured the effect of monthly income or wealth quantile on frailty. Our data showed that monthly income and wealth quantile could not significantly change the prevalence of frailty (Table 3). After adjusting for several factors, multivariate logistic regression revealed that low physical activity [OR 31.73 (18.44–54.60)], cancer [OR 3.28 (1.27–8.44)], depression [OR 2.42 (1.97–2.98)], age [OR 1.11 (1.08–1.14)], waist circumference [OR 1.03 (1.01–1.06)], BMI [OR 1.07 (1.01–1.14)], MNA score [OR 0.85 (0.79–0.92)], polypharmacy [OR 2.26 (1.30–3.95)] and male gender [OR 0.63 (0.42–0.93)] were independently associated with frailty. WBC, smoking, marital status, and number of comorbidities were not independently associated with frailty (Table 4).

**Table 3:** The prevalence of frailty and pre-frailty in different ranges of monthly income.

Income range	Less than 100 USD	100–200 USD	200–500 USD	500–1000 USD	1000–2000 USD	More than 2000 USD
Robust	148 (44.71%)	105 (42.86%)	556 (41.90%)	186 (43.76%)	11 (32.35%)	10 (37.04%)
Pre-frail	146 (44.11%)	119 (48.57%)	637 (48.00%)	196 (46.12%)	20 (58.82%)	14 (51.85%)
Frail	37 (11.18%)	21 (8.57%)	134 (10.10%)	43 (10.12%)	3 (8.82%)	3 (11.11%)

**Table 4:** Binary logistic regression reporting ORs and 95% CI for the association of different variables with frailty.

Model	Variable		OR with 95% CI	*P* value	Variable	OR with 95% CI	*P* value
Univariate logistic regression	Age		1.10 (1.08–1.12)	0.000	Low physical activity	4.21 (3.27–5.41)	0.000
	Male gender		0.28 (0.21–0.36)	0.000	Cancer	2.60 (1.30–5.22)	0.007
	Marital status	Married	0.84 (0.19–3.70)	0.813	MNA score	0.67 (0.64–0.71)	0.000
		Divorced	0.82 (0.10–6.62)	0.855	PHQ-9 score	3.68 (3.18–4.25)	0.000
		Widow	2.63 (0.59–11.74)	0.204	Waist circumference	1.02 (1.01–1.03)	0.000
	Drug number	1–4 drugs	1.32 (0.79–2.18)	0.285	BMI	1.06 (1.04–1.08)	0.000
		Polypharmacy	3.65 (2.38–5.59)	0.000	Smoking	0.75 (0.55–1.02)	0.069
	WBC count		1.02 (0.97–1.07)	0.501	Comorbidity number	2.43 (2.08–2.84)	0.000
Multivariate logistic regression	Age		1.11 (1.08–1.14)	0.000	Low physical activity	31.73 (18.44–54.60)	0.000
	Male gender		0.63 (0.42–0.93)	0.021	Cancer	3.28 (1.27–8.44)	0.014
	Marital status	Married	1.75 (0.19–15.65)	0.619	MNA score	0.85 (0.79–0.92)	0.000
		Divorced	0.71 (0.03–15-47)	0.827	PHQ-9 score	2.42 (1.97–2.98)	0.000
		Widow	2.28 (0.25–20.48)	0.463	Waist circumference	1.03 (1.01–1.06)	0.016
	Drug number	1–4 drugs	0.99 (0.52–1.88)	0.98	BMI	1.07 (1.01–1.14)	0.036
		Polypharmacy	2.26 (1.30–3.95)	0.004	Smoking	0.69 (0.46–1.04)	0.076
	WBC count		1.02 (0.95–1.10)	0.922	Comorbidity number	0.99 (0.77–1.27)	0.922

Age, gender, physical activity, cancer, MNA score, PHQ-9 score, waist circumference, BMI, WBC, smoking, marital status, number of comorbidities, and polypharmacy were entered into the multivariate logistic regression model.

Pseudo R-Squared (R^2^) = 0.4628 and log likelihood: -421.9934.

Only 70.99% (95% CI 69.18–72.24%) of participants were independent based on Katz activities of daily living (ADL). The independence ratio in Katz ADL was markedly higher in males than in females (81.02% vs. 61.62%). However, dependency in ≥ 4 domains was found in less than 1% of participants (Table 5). Instrumental activities of daily living (IADL) showed a lower prevalence of independence (40.83%, 95% CI 38.87–40.81). The prevalence of dependence increased with the age of participants, and no woman ≥ 85 years old was independent (Supp Table 3).

**Table 5:** The prevalence of dependency in ADL-Katz function according to the number of domains and sex.

Gender	Age groups	Without dependency (%)			Dependent in one domain (%)			Dependent in 2–3 domains (%)			Dependent in ≥ 4 domains (%)		
Females	60–64 years	67.63	62.12	72.69	31.41	26.37	36.93	0.64	0.16	2.55	0.32	0.05	2.24
	65–69 years	64.30	60.18	68.22	34.74	30.81	38.89	0.58	0.19	1.76	0.38	0.10	1.53
	70–74 years	61.58	54.60	68.12	35.59	29.12	42.64	2.26	0.85	5.90	0.56	0.08	3.93
	75–79 years	54.20	45.93	62.24	40.46	32.54	48.91	3.82	1.61	8.80	1.53	0.38	5.96
	80–84 years	50.75	39.34	62.08	43.28	32.47	54.78	4.48	1.44	13.08	1.49	0.21	9.96
	≥ 85 years	47.50	32.19	63.29	35.00	21.41	51.56	10.00	3.79	23.84	7.50	2.41	21.01
	All age-groups*	61.76	58.92	64.53	35.10	32.40	37.90	2.11	1.36	3.25	1.03	0.54	1.94
	All age groups**	61.62	58.82	64.35	35.25	32.59	38.02	2.12	1.38	3.24	1.01	0.53	1.89
Males	60–64 years	90.07	86.07	93.01	9.57	6.68	13.55	0.00	0.00	0.00	0.35	0.05	2.47
	65–69 years	82.70	78.76	86.04	16.59	13.31	20.48	0.47	0.12	1.87	0.24	0.03	1.68
	70–74 years	78.28	71.90	83.55	20.71	15.45	27.18	0.51	0.07	3.45	0.51	0.07	3.48
	75–79 years	72.67	65.62	78.74	24.67	18.60	31.93	0.67	0.10	4.49	2.00	0.66	5.94
	80–84 years	65.79	54.44	75.58	31.58	22.16	42.81	1.32	0.18	8.78	1.32	0.18	8.81
	≥ 85 years	71.43	52.15	85.15	21.43	9.88	40.42	3.57	0.50	21.53	3.57	0.50	21.53
	All age groups*	81.00	78.65	83.14	17.54	15.44	19.85	0.61	0.24	1.54	0.85	0.40	1.79
	All age groups**	81.02	78.77	83.07	17.59	15.57	19.82	0.59	0.24	1.40	0.81	0.39	1.65
Total Participants	60–64 years	78.28	74.59	81.57	21.04	17.76	24.75	0.34	0.08	1.34	0.34	0.08	1.34
	65–69 years	72.53	69.59	75.30	26.62	23.85	29.58	0.53	0.22	1.25	0.32	0.10	0.98
	70–74 years	70.40	65.50	74.87	27.73	23.27	32.69	1.33	0.56	3.15	0.53	0.13	2.11
	75–79 years	64.06	58.72	69.07	32.03	27.03	37.47	2.14	0.97	4.63	1.78	0.74	4.19
	80–84 years	58.74	50.44	66.57	37.06	29.42	45.41	2.80	1.05	7.25	1.40	0.35	5.40
	≥ 85 years	57.35	44.75	69.07	29.41	19.54	41.69	7.35	3.12	16.37	5.88	2.21	14.74
	All age groups*	71.25	69.43	73.01	26.44	24.71	28.24	1.37	0.92	2.03	0.94	0.58	1.52
	All age groups**	70.99	69.18	72.74	26.72	24.99	28.53	1.36	0.92	1.99	0.93	0.58	1.48

*Age and sex standardized based on the Iranian population in 2016.

**Age-standardized based on World Health Organization Population 2000–2025.

Among all participants, 27.40% (95% CI 25.68–29.20) had urinary incontinence. The prevalence of urinary incontinence in women was more than twice that in men (36.44% vs. 17.65%). In addition, the prevalence of urinary incontinence among women increased with age from 31% in those with 60–64 years of age to 45% in those ≥ 85 years of age. However, the prevalence of fecal incontinence was less than 1% among all participants, which was similar in both sexes (Supp Table 4).

WHO age-standardized prevalence of immobility was 5.32% in all participants. The prevalence of immobility was three-time higher in women than in men, and it increased with age (Table 6).

**Table 6:** The age-standardized prevalence of immobility in older population.

Variables	Total Participants (%) N = 2392			Female (%) N = 1235			Male (%) N = 1157		
Age groups	Point estimation (95% CI)			Point estimation (95% CI)			Point estimation (95% CI)		
60–64 years	2.18	1.27	3.72	3.50	1.94	6.24	0.71	0.18	2.78
65–69 years	2.42	1.60	3.65	3.44	2.18	5.38	1.17	0.50	2.72
70–74 years	5.56	3.61	8.46	8.43	4.96	13.97	3.00	1.37	6.46
75–79 years	10.64	7.44	14.99	16.67	11.15	24.16	5.33	2.71	10.25
80–84 years	5.48	2.75	10.62	8.70	3.92	18.19	2.60	0.65	9.84
≥ 85 years	23.53	14.94	35.02	32.50	20.01	48.09	10.34	3.34	27.81
All age groups *	5.26	4.36	6.33	7.91	6.39	9.76	2.55	1.70	3.80
All age groups **	5.32	4.42	6.40	8.00	6.48	9.86	2.50	1.71	3.67

*Age and sex standardized based on the Iranian population in 2016.

**Age-standardized based on World Health Organization Population 2000–2025.

WHO age-standardized prevalence of mild depression, moderate depression, and severe depression was 16.48%, 5.61%, and 4.17%, respectively. The prevalence of depression was not statistically significant in males but it was prominent in females. There was a considerable difference between males and females regarding depression. This difference was notable in severe depression and it was several times more prevalent in females than males (Supp Table 5).

As mentioned, the cognitive function was assessed using the Mini-cog tool. WHO age-standardized prevalence of cognitive dysfunction was 53.74% (95% CI 51.70–55.77%) in all participants. The prevalence of cognitive impairment increased with age. Cognitive impairment was more common in females and this difference was more prominent in younger participants than in older participants (Supp Table 4).

Mild malnutrition was assessed using the Mini Nutritional Assessment (MNA). Only 1.62% of subjects had mild malnutrition and this problem was nearly three times more common in females than in males (2.35% vs. 0.82%). There was not a linear relationship between mild malnutrition and age, but the highest prevalence of mild malnutrition was observed in participants ≥ 85 years of age (5.88%) (Supp upp Table 4).

**Figure 1:**
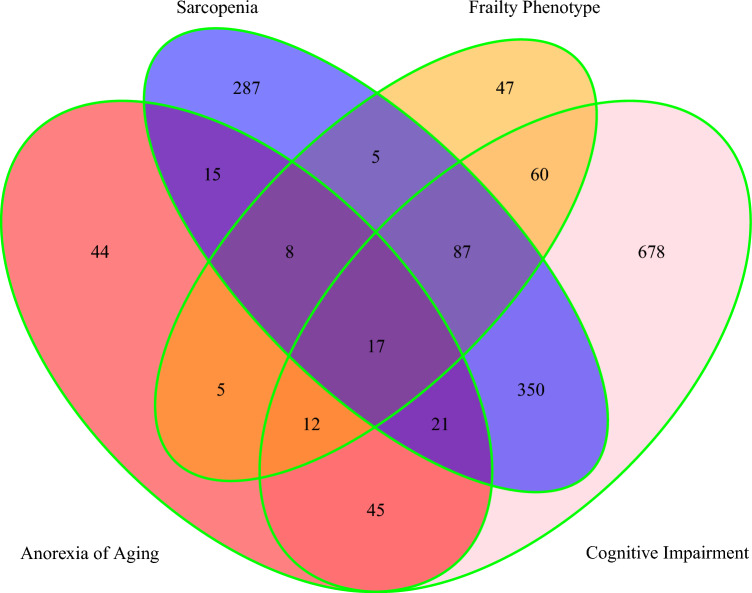
Venn diagram depicting the coexistence of frailty, sarcopenia, cognitive impairment, and anorexia of aging.

In addition, we measured the co-incidence of 4 components of geriatric giants including frailty, sarcopenia, cognitive impairment, and anorexia. Among all participants in this study, 725 (31.14%) participants had none of these components, 978 (42.01%) participants had one of these components, 482 (20.70%) participants had two of these components, 126 (5.41%) participants had three of these components, and 17 (0.73%) participants had four of these components (Fig. 1). Multinomial logistic regression also indicated that male gender, MNA score, and BMI were negatively associated with the presence or coexistence of frailty, sarcopenia, cognitive impairment, and anorexia of aging, whereas age, waist circumference, and low physical activity were positively associated with the presence or coexistence of frailty, sarcopenia, cognitive impairment, and anorexia of aging. In particular, low physical activity showed the greatest relative risk ratios (RRRs) for the co-existence of geriatric giants. Moreover, cancer, smoking, alcohol consumption, WBC count, marital status, and polypharmacy were not significantly associated with the coexistence of frailty, sarcopenia, cognitive impairment, and anorexia of aging (Table 7).

**Table 7:** Multinomial logistic regression reporting the RRR and 95% CI for the association of different variables with the coexistence of frailty, sarcopenia, cognitive impairment, and anorexia of aging.

Variables		None of criterion existed	One criterion vs. None of criterion	Two criteria vs. None criterion	Three criteria vs. None criterion	Four criteria vs. None criterion
Age		Reference group	1.09 (1.06–1.11)	1.15 (1.12–1.18)	1.21 (1.16–1.25)	1.22 (1.12–1.33)
Sex		Reference group	0.46 (0.36–0.59)	0.34 (0.25–0.47)	0.36 (0.20–0.64)	0.36 (0.08–1.65)
Low physical activity		Reference group	1.93 (1.26–2.94)	2.55 (1.62–4.01)	15.57 (7.86–30.83)	98.11 (8.97–1072.75)
Cancer		Reference group	1.08 (0.40–2.97)	1.64 (0.57–4.69)	0.91 (0.20–4.14)	NA
MNA score		Reference group	0.80 (0.74–0.86)	0.67 (0.61–0.73)	0.57 (0.51–0.64)	0.39 (0.31–0.49)
PHQ-9 score		Reference group	0.86 (0.72–1.03)	1.05 (0.85–1.30)	1.48 (1.11–1.97)	1.66 (0.92–3.01)
Waist circumference		Reference group	1.02 (1.01–1.04)	1.03 (1.01–1.05)	1.06 (1.02–1.10)	1.05 (0.95–1.16)
BMI		Reference group	0.93 (0.89–0.97)	0.81 (0.76–0.85)	0.82 (0.74–0.91)	0.94 (0.70–1.26)
Smoking		Reference group	1.15 (0.89–1.50)	1.09 (0.78–1.52)	0.96 (0.55–1.70)	1.24 (0.30–5.09)
WBC		Reference group	1.00 (0.95–1.05)	0.98 (0.92–1.04)	1.00 (0.91–1.10)	1.03 (0.78–1.36)
Alcohol consumption		Reference group	1.42 (0.61–3.30)	0.34 (0.06–1.85)	NA	NA
Single		Reference group	1	1	1	1
Marital status	Married	Reference group	1.14 (0.33–3.92)	1.18 (0.24–5.79)	1.16 (0.09–14.69)	NA
	Divorced	Reference group	0.53 (0.10–2.72)	0.66 (0.09–5.10)	NA	NA
	Widow	Reference group	1.20 (0.34–4.21)	1.24 (0.25–6.20)	1.05 (0.08–13.59)	NA
	No drug consumed	Reference group	1	1	1	1
Number of drugs	1–4 drugs consumed	Reference group	0.98 (0.71–1.34)	0.73 (0.48–1.10)	0.58 (0.28–1.21)	0.20 (0.03–1.46)
	5 drugs or more consumed (Polypharmacy)	Reference group	0.84 (0.62–1.13)	0.67 (0.46–0.98)	0.56 (0.29–1.07)	0.27 (0.06–1.28)

NA: could not be assessed by the model. Age, sex, cancer, physical activity, number of drugs, marital status, BMI, waist circumference, WBC, smoking, MNA score, PHQ-9 score, and alcohol consumption were entered into the model. Log likelihood =  − 2350.773, Pseudo R^2^ = 0.1876.

## Discussion

In this study, we found that the prevalence of frailty and pre-frailty was 12.07% and 46.76% in Southern Iran, respectively. Frailty, cognitive impairment, dependence, urinary incontinence, immobility, depression, anorexia, and malnutrition were considerably more common in older ages and among women. Low physical activity, cancer, female gender, malnutrition, depression, age, waist circumference, and BMI were positively and independently associated with frailty. Among them, low physical activity was the strongest predictor of frailty in this study.

As mentioned previously, each component of geriatric giants is associated with other components^5^. For instance, data from 5474 individuals ≥ 70 years of age revealed that urinary incontinence in older age is significantly and positively associated with falls^18^. Reciprocally, falling was associated with urinary incontinence and physical limitation^18^. Consistently, we have shown that malnutrition/anorexia, depression, and low physical activity are associated with frailty. Thus, preventing or treating one component of geriatric giants may improve the others.

The Longitudinal Ageing Study in India (LASI) with 31,464 participants reported that among patients aged ≥ 60, late-life depression and cognitive impairment had an overall prevalence of 0.7% and 13.7%, respectively^19^. Furthermore, late-life depression was 74% and 69% more common among those who had difficulty with ADL and IADL^19^. Moreover, the study reported that cognitive impairment was more frequent among depressed individuals^19^. These findings may partly warrant the higher prevalence of cognitive impairment among females in our study. As depression was more common among women in all ranges of age, cognitive impairment followed the same pattern. In particular, the prevalence of cognitive impairment was markedly higher in women aged 60–74 years, compared with men of the same age. In addition to a higher prevalence of depression, low physical activity may lead to a higher prevalence of cognitive impairment among women. A recent study has shown that moderate-intensity physical activity can substantially improve cognitive function and quality of life in those with mild cognitive impairment^20^.

A systematic review of 23 articles indicated that isolated fecal incontinence was found in 3.5% (interquartile range 2.8%) of older people who resided in care homes^21^. Besides, cognitive impairment, urinary incontinence, advanced age, limited functional capacity, reduced mobility, and diarrhea were correlated with fecal incontinence in this study^21^. Fecal incontinence was less common in our study as our participants had better health conditions.

The prevalence of frailty is expected to grow in the aging population^22^. Frailty is associated with a marked decline in daily functioning and significantly increased susceptibility to stressors, falls, hospitalization, and mortality^22^. Previous studies have shown that frailty is positively correlated with disability-adjusted life years (DALY); therefore, frail patients constitute a substantial proportion of the global burden of disease^23^. Although many studies unveiled the indispensable importance of frailty, still there is little progress in its management^22,24^. In particular, having better knowledge about the risk factors of frailty can help to modify healthcare services and prevent frailty or its complications through early intervention^22^. With the increase in life expectancy in the coming decades, years spent with frailty will gradually increase, which is anticipated to substantially escalate health care expenses^24^.

Similar to our findings, a meta-analysis including 81,258 subjects revealed that the pooled prevalence of pre-frailty and frailty were 43% and 10%, respectively^11^. In addition, the pooled prevalence of frailty was 8% based on the Fried frailty phenotype^11^. Consistent with our findings, the prevalence of frailty exponentially increased with age in this meta-analysis^11^. After adjusting for other factors among 484 participants, Gobbens et al*.*^25^ reported that medium income, an unhealthy lifestyle, and multimorbidity can predict frailty. They also reported that age was only associated with physical frailty, history of life events was only associated with psychological frailty, and female gender was only associated with social frailty^25^. A study with 1867 participants aged ≥ 65 years indicated that only age and education were associated with frailty^26^. A recent study from China with 14,314 participants reported that older age, being a professional or technician before 60 years of age, poor economic condition, and poor oral hygiene are risk factors for frailty, while eating rice as a staple food, regular exercise, having a spouse as the first person to share thoughts with, doing an annual physical examination, and not needing a caregiver during illness are protective factors against frailty^27^. Furthermore, the meta-analysis of 14 studies uncovered that increasing age, female gender, activities of daily living disability, and having three or more chronic diseases are positively associated with frailty^11^. We showed that low physical activity, cancer, female gender, malnutrition, depression, age, waist circumference, polypharmacy, and BMI were positively and independently associated with frailty. However, WBC, smoking, and marital status were not independently associated with frailty in this study.

Low physical activity can be both a risk factor and an outcome of frailty^28^. Frail patients become physically limited and rely on their caregivers and family members for their daily functioning^29^. Even, it has been observed that frail patients have specific patterns of walking^29^. On the other hand, physical dependence and the absence of physical activity make individuals frail^28^. On the contrary, maintaining a minimum level of physical activity, particularly aerobic exercise, may prevent or postpone frailty in older age^28^. Herein, a study including 780 patients unveiled that age, malnutrition, functional dependence, and severe knee osteoarthritis can enhance the risk of frailty^28^. Among the risk factors, severe knee osteoarthritis, which utterly restricts physical activity, was associated with an 18-time higher risk of frailty. However, mild and moderate osteoarthritis that allow a sufficient level of physical activity for daily functioning were not associated with frailty^28^.

A systematic review of 20 studies with 2916 patients revealed that frailty and pre-frailty are frequently observed among cancer patients (median prevalence of 42% and 43%, respectively)^30^. In addition, frailty predicts higher rates of mortality and treatment-associated complications in cancer patients^30^.

Senescence is associated with altered biological processes and metabolism, ineffective immune response, and impaired or weak response to stressors, which may interfere with normal homeostasis^31–33^. Consistently, it has been unfolded that abnormal immune response, deregulation of cellular metabolism, and impaired metabolism of macromolecules are involved in the pathogenesis of frailty^34^. Similar o frailty, aging is accompanied by several chronic diseases and comorbidities resulting in decreased functional reserve^35^. Thus, a deeper insight into the biology of aging may improve the prevention, screening, and treatment of frailty^31^.

Similar to our study, previous studies showed that being female can significantly elevate the risk of frailty, which may be due to physiologic differences in metabolism, muscle mass, body composition, mental status, physical strength, and life expectancy^36,37^. Furthermore, it has been observed that frail women have longer survival than both frail and non-frail men^37^. Interestingly, by stratifying participants into different groups of BMI, Hubbard et al*.* unveiled that participants with a BMI of 25–29.9 are at the lowest risk of frailty, but a BMI of higher or lower than this range is associated with a greater risk of frailty^38^. In particular, those with a BMI of less than 20 were at the greatest risk of frailty^38^. Likewise, a meta-analysis by Yuan et al. indicated that both being underweight or obese was associated with an increased risk of frailty^39^. Consistently, our findings indicated that both high BMI and malnutrition are independently associated with frailty.

There are some limitations to this study. First, the prevalence of frailty, pre-frailty, and other components of geriatric giants cannot be applied to other regions, as none of the previous or future studies can do the same because the prevalence is specific to each region and its population. However, the associations found in this study can be validated by other studies in different regions and populations. Second, due to the cross-sectional nature of this study, it cannot support causality and future prospective studies are needed to measure the presence of causality in the observed relationship. Third, large studies with participants from different regions are needed to better reflect the prevalence of geriatric giants in Iran and the Middle East.

With all shortcomings, compared with previous studies^12,40,41^, this study has some strengths. First, the greater sample size and better sampling method in this study can help better reflect the prevalence of geriatric giants in the study region. Second, most studies from Iran focused on frailty, but this study is the first study that measured different components of geriatric giants in the Iranian population. Third, this study comprehensively assessed the associations between geriatric giants or frailty and different variables, which can provide new ideas for future studies and improve the screening and prevention of frailty.

## Conclusion

Our findings showed that the prevalence of frailty and pre-frailty was 12.07% and 46.76% in a sample from the Iranian population. We found that the prevalence of frailty exponentially increases in older ages. Frailty and other giants of geriatrics such as cognitive impairment, dependence, urinary incontinence, immobility, depression, anorexia, and malnutrition were markedly more common in women than in men. Low physical activity, cancer, female gender, malnutrition, depression, age, waist circumference, polypharmacy, and BMI were positively and independently associated with frailty. Among them, low physical activity was the strongest predictor of frailty. Our findings provide a deeper insight into the prevention of frailty and can be useful for the design of future interventional studies.

## Methods

### Participants

We used data from the second wave of the Bushehr Elderly Health Program (BEHP). Participants were urban residents from Bushehr in the south of Iran. They were included using a multistage cluster random sampling method. The detail of the first and second wave of this study was published elsewhere^17^. In the first wave, 3000 older adults (age > 60 years) who lived in a community in Bushehr were enrolled. The participants were chosen from 75 separate neighborhoods defined by the local municipality. The number of participants selected from each neighborhood was proportional to the number of households registered in the last census. In the second wave, 2480 subjects from the first wave were enrolled again. They were invited to a health center, and their health status was evaluated. Data on demographic characteristics, lifestyle factors, general health, medical history, and mental and functional status were collected through a questionnaire. Assessment of anthropometric indices, performance-based tests, muscle strength tests, blood pressure evaluation, and body composition measurements were performed. Because of incomplete data, finally, 2392 subjects were included for analysis. The reason for incomplete data was death or insufficient compliance of patients for completing the assessments.

### Data collection

The phenotype of frailty was defined according to the Fried phenotype. Unintentional weight loss ≥ 4.5 kg during the past year was asked. Exhaustion was defined as positive answers to two questions of the Center for Epidemiological Studies Depression (CES-D). Slowness was defined based on sex and height. Handgrip strength and physical activity were determined using the lowest quintile of mean handgrip measures after three times of measurements in each hand. Low physical activity was defined as the lowest metabolic equivalent during 1 week based on WHO physical activity questionnaire. The presence of one or two of the aforementioned criteria was defined as pre-frailty and the presence of more than two criteria was defined as frailty^42^.

Demographic data were also collected during the interview. Anthropometric data such as weight, height, and waist circumference were measured based on NHANCE-3 guidelines. Body mass index (BMI) was calculated by dividing weight (kg) by squared height (meter). Overnight fasting blood samples were collected and fasting blood sugar, Hb A1C, and complete blood count (CBC) were measured using automatic standardized devices. Diabetes mellitus and hypertension were defined according to ADA 2018 and JNC-8 criteria.

Urinary and fecal incontinence was defined as the incidence of any episode of incontinence during the last 6 months. Mobility and instability were evaluated by asking the participants and their partners about their ability to move. In addition, timed up and go test and short physical performance battery (SPPB) scores were assessed^43^. The incidence and number of falls during the last year were recorded. Anorexia in the past 3 months was evaluated by the Mini Nutritional Assessment (MNA) questionnaire^44^. Furthermore, information regarding drug history and significant weight loss (≥ 4.5 kg) during the last year was also collected. Taking more than five drugs per day was considered polypharmacy.

Katz ADL instrument was utilized for assessing independence in basic daily activities, including bathing, feeding, urinary and fecal continence, dressing, toileting, and transferring^45^. Lawton instrumental activity of daily living was used for assessing intermediate daily function. This instrument has eight domains. A score of eight shows independence in all areas and a score of zero shows complete dependence.

Mood was measured using patient health questionnaire 9 (PHQ-9)^46^. This questionnaire has nine items and each item is a Likert-type question with a score from zero to three. The maximum score is 27 showing the worst mood and zero showing the best mood. The validity and reliability of this tool were approved in the Iranian population^47^.

Cognitive function was assessed using two instruments; the Mini-Cog score and the category fluency test. Mini-Cog had two stages^48^. In the first stage, the ability to recall three words was evaluated. Remembering all words was considered normal cognition and recalling none of them was considered impaired cognition. Participants who recalled one or two words, entered the second stage, a clock drawing test. Those who correctly drew the clock were considered cognitively intact; otherwise, the subjects were considered cognitively impaired. Furthermore, the category fluency test was used for naming animals. Subjects were categorized into impaired cognition and normal cognition groups based on the number of years they spent in school. Participants with impaired cognition in at least one test were categorized into the impaired cognition group.

We also measured the frailty index comprising 35 items. These items were needing assistance with bathing, dressing, getting in and out of a chair, walking around the house, eating, grooming, using toilet, going up and down stairs, shopping, housework, meal preparation, taking medication, managing finances, experiencing weight loss of more than 4.5 kg in the last year, walking outside, feeling that everything is an effort, feeling depressed or lonely, having trouble getting going, high blood pressure, heart attack, congestive heart failure, stroke, cancer, diabetes, arthritis, chronic lung disease, chronic kidney disease, gait speed, BMI, grip strength, cognitive impairment (Mini-Cog), seizures, urinary incontinence, and bowel incontinence.

The frailty index did not include peak flow, shoulder strength, and timed normal pace due to the lack of clear cut-points in the original article^49^.

Recorded procedures were used to transform categorical, ordinal, and interval variables into a common scale ranging from 0 to 1. In this mapping, 0 represents the absence of a deficit, while 1 represents the full presence of the deficit. Individual deficit scores were combined to create an index, where a score of 0 indicated the absence of deficits and a score of 1 indicated the presence of all 40 deficits^49^. Although we report the prevalence of frailty based on two measures, frailty index and Fried frailty phenotype, the latter was used for constructing regression models and interpreting the associations between frailty and other variables.

All questionnaires used in this study were previously translated into Persian language and validated in Iran^17^.

### Ethics approval

The Ethics Committee of Tehran University of Medical Sciences and Bushehr University of Medical Sciences approved the protocol of this study (IR.TUMS.EMRI.REC.1394.0036). Written informed consent was obtained before participation. The protocol of this study followed the Ethics standards defined by the 2013 version of the Declaration of Helsinki. This study has been reported following the STROBE Statement.

### Statistical analysis

The prevalence of variables was calculated as a survey analysis with the weighting of the Iranian population census 2016. For comparing our findings with those from other countries, the data were age-standardized based on the World Health Organization (WHO) population 2000–2025.

Quantitative variables are expressed as mean (SD). The prevalence of pre-frailty, frailty, and other components of geriatric giants was assessed in males and females and across different ranges of age. We also used the Chi-square (χ^2^) test to assess the effect of monthly income or wealth quantile on the prevalence of frailty.

A binary logistic regression model was used to identify the independent factors associated with frailty. The following variables were adjusted in the binary logistic regression model: age, gender, physical activity, cancer, MNA score, PHQ-9 score, waist circumference, BMI, WBC, smoking, marital status, number of comorbidities, and polypharmacy. The presence of the following comorbidities was assessed to calculate the number of comorbidities: hypertension, diabetes, hypo- and hyperthyroidism, chronic kidney disease, rheumatoid arthritis, osteoarthritis, Alzheimer’s disease, liver disease, lung disease, epilepsy, and Parkinson’s. We also used multinomial logistic regression model to investigate the associations between the number of geriatric giants and age, sex, low physical activity, cancer, MNA score, PHQ-9 score, waist circumference, BMI, and smoking.

In addition, we measured the co-incidence of 4 components of geriatric giants, including frailty, sarcopenia, cognitive impairment, and anorexia. Moreover, a multinomial logistic regression model was developed to assess the effect of different variables on the co-existence of frailty, sarcopenia, cognitive impairment, and anorexia. Age, sex, physical activity, cancer, MNA score, PHQ-9 score, waist circumference, BMI, smoking, WBC count, alcohol consumption, marital status, and the number of drugs were entered into this analysis as independent variables.

Stata package version 12 (StataCorp Texas, USA) was utilized for analysis. *P* values < 0.05 were considered statistically significant.

## Supplementary Information

Supplementary Tables.

## Data Availability

Data analyzed for this article will be provided by the corresponding authors upon a reasonable request.
