# Supplementary material for: The prevalence and predictors of geriatric giants in community-dwelling older adults: a cross-sectional study from the Middle East
Source: Sci Rep. 2023 Jul 31;13:12401. doi: 10.1038/s41598-023-39614-4 (PMC10390524; doi:10.1038/s41598-023-39614-4)
Supplement: Supplementary file 1 — Supplementary Tables. [file 41598_2023_39614_MOESM1_ESM.docx]

**Supplementary table 1. The prevalence of frailty based on frailty index according to age groups and genders.**

| **Variables** | **Total Participants (%)** | | **Female (%)** | | **Male (%)** | |
| --- | --- | --- | --- | --- | --- | --- |
|  | **Pre-frailty** | **Frailty** | **Pre-frailty** | **Frailty** | **Pre-frailty** | **Frailty** |
| **Age groups** | **Point estimation**  **(95% CI)** | **Point estimation**  **(95% CI)** | **Point estimation**  **(95% CI)** | **Point estimation**  **(95% CI)** | **Point estimation**  **(95% CI)** | **Point estimation**  **(95% CI)** |
| **60 – 64 years** | 61.66 (57.58-65.58) | 7.59 (5.67-10.09) | 70.57 (65.14-75.47) | 12.37 (9.09-16.62) | 51.69 (45.68-57.64) | 2.24 (1.01-4.91) |
| **65 – 69 years** | 68.11 (64.99-71.08) | 10.33 (8.50-12.50) | 76.78 (72.83-80.31) | 14.66 (11.80-18.08) | 57.70 (52.85-62.41) | 5.13 (3.37-7.75) |
| **70 – 74 years** | 62.50 (57.37-67.36) | 16.94 (13.41-21.19) | 66.67 (59.18-73.40) | 27.38 (21.15-34.64) | 58.85 (51.74-65.62) | 7.81 (4.76-12.57) |
| **75 – 79 years** | 66.16 (60.21-71.64) | 22.81 (18.12-28.29) | 60.98 (52.06-69.22) | 35.77 (27.77-44.65) | 70.71 (62.62-77.68) | 11.43 (7.11-17.87) |
| **80 – 84 years** | 69.40 (61.06-76.64) | 26.12 (19.36-34.24) | 57.38 (44.65-69.19) | 42.62 (30.81-55.35) | 79.45 (68.58-87.26) | 12.33 (6.51-22.12) |
| **≥ 85 years** | 39.66 (27.87-52.76) | 55.17 (42.20-67.48) | 32.35 (18.74-49.80) | 67.65 (50.20-81.26) | 50.00 (30.62-69.37) | 37.50 (20.50-58.27) |
| **All age groups** | 64.75 (62.76-66.69) | 14.20 (12.83-15.69) | 69.81 (67.12-72.37) | 21.09 (18.84-23.51) | 59.37 (56.44-62.23) | 6.88 (5.52-8.53) |

**Supplementary table 2. The prevalence of the features of frailty phenotype according to age groups and genders.**

| **Variables** | | **Total Participants (%)**  **N = 2392** | **Female (%)**  **N = 1235** | **Male (%)**  **N = 1157** |
| --- | --- | --- | --- | --- |
| **Total Participants** | **Weight loss** | 6.15 (5.25 – 7.18) | 8.82 (7.40 – 10.49) | 3.26 (2.39 – 4.33) |
|  | **Exhaustion** | 18.53 (17.04 – 20.13) | 28.49 (25.85 – 31.29) * | 8.28 (6.69 – 10.21) * |
|  | **Low PA** | 21.65 (20.16 – 23.21) * | 21.83 (19.68 – 24.14) | 21.48 (19.36 – 23.77) |
|  | **Low HG** | 19.64 (18.19 – 21.17) | 19.39 (17.36 – 21.61) | 19.90 (17.81 – 22.16) |
|  | **Low walking speed** | 34.16 (32.32 – 36.05) | 47.46 (44.67 – 50.26) | 19.81 (17.64 – 22.17) |
| **60 – 64 years** | **Weight loss** | 6.02 (4.29 – 8.39) | 9.87 (6.87 – 13.98) | 1.76 (0.73 – 4.18) |
|  | **Exhaustion** | 16.05 (13.27 – 19.29) | 24.20 (19.78 – 29.25) | 7.04 (4.65 – 10.53) |
|  | **Low PA** | 8.70 (6.64 – 11.32) | 6.37 (4.13 – 9.70) | 11.27 (8.01 – 15.63) |
|  | **Low HG** | 8.36 (6.43 – 10.80) | 9.87 (7.04 – 13.68) | 6.69 (4.40 – 10.05) |
|  | **Low walking speed** | 22.07 (19.03 – 25.44) | 31.85 (26.84 – 37.31) | 11.27 (8.09 – 15.48) |
| **65 – 69 years** | **Weight loss** | 6.42 (5.01 – 8.19) | 8.00 (5.94 – 10.68) | 4.46 (2.87 – 6.87) |
|  | **Exhaustion** | 18.84 (16.49 – 21.44) | 28.05 (24.44 – 31.98) | 7.51 (5.33 – 10.49) |
|  | **Low PA** | 12.32 (10.29 – 14.65) | 10.28 (7.95 – 13.20) | 14.79 (11.51 – 18.80) |
|  | **Low HG** | 12.93 (11.01 – 15.13) | 12.76 (10.24 – 15.80) | 13.14 (10.43 – 16.44) |
|  | **Low walking speed** | 28.39 (25.60 – 31.36) | 40.95 (36.82 – 45.22) | 12.91 (10.09 – 16.37) |
| **70 – 74 years** | **Weight loss** | 6.08 (4.11 – 8.92) | 10.11 (6.56 – 15.27) | 2.50 (1.03 – 5.94) |
|  | **Exhaustion** | 18.78 (15.14 – 23.06) | 32.58 (25.88 – 40.08) | 6.50 (3.88 – 10.69) |
|  | **Low PA** | 20.11 (16.16 – 24.73) | 23.59 (17.87 – 30.47) | 17.00 (12.35 – 22.93) |
|  | **Low HG** | 22.49 (18.82 – 26.63) | 24.71 (19.28 – 31.10) | 20.50 (15.65 – 26.38) |
|  | **Low walking speed** | 38.62 (33.79 – 43.69) | 58.43 (50.86 – 65.62) | 21.00 (16.02 – 27.03) |
| **75 – 79 years** | **Weight loss** | 6.03 (3.71 – 9.64) | 9.09 (5.11 – 15.66) | 3.33 (1.38 – 7.85) |
|  | **Exhaustion** | 18.79 (14.62 – 21.43) | 28.03 (20.97 – 36.37) | 10.67 (6.50 – 17.02) |
|  | **Low PA** | 39.36 (33.86 – 45.15) | 43.94 (35.36 – 52.90) | 35.33 (28.09 – 43.31) |
|  | **Low HG** | 37.59 (32.35 – 43.13) | 40.15 (32.22 – 48.63) | 35.33 (28.24 – 43.13) |
|  | **Low walking speed** | 49.29 (43.45 – 55.15) | 66.66 (58.24 – 74.14) | 34.00 (26.89 – 41.91) |
| **80 – 84 years** | **Weight loss** | 6.16 (3.23 – 11.45) | 10.14 (4.88 – 19.90) | 2.60 (0.64 – 9.82) |
|  | **Exhaustion** | 19.86 (14.29 – 26.93) | 30.43 (20.92 – 41.98) | 10.39 (5.28 – 19.42) |
|  | **Low PA** | 50.00 (42.17 – 57.82) | 59.42 (47.79 – 70.08) | 41.56 (31.16 – 52.77) |
|  | **Low HG** | 48.63 (40.58 – 56.75) | 37.68 (27.12 – 49.55) | 58.44 (46.98 – 69.06) |
|  | **Low walking speed** | 61.64 (53.80 – 68.92) | 79.71 (69.35 – 87.21) | 45.45 (34.78 – 56.56) |
| **≥ 85 years** | **Weight loss** | 4.41 (1.47 – 12.46) | 2.50 (0.35 – 15.77) | 6.90 (1.79 – 23.08) |
|  | **Exhaustion** | 30.88 (20.87 – 43.08) | 42.50 (28.78 – 57.48) | 14.28 (5.43 – 32.59) |
|  | **Low PA** | 82.35 (70.71 – 88.48) | 92.50 (79.57 – 97.50) | 65.52 (48.19 – 79.51) |
|  | **Low HG** | 59.42 (47.91 – 69.98) | 57.50 (42.18 – 71.50) | 62.07 (44.85 – 76.70) |
|  | **Low walking speed** | 73.91 (62.55 – 82.77) | 87.50 (73.01 – 94.76) | 55.17 (38.41 – 70.84) |

**Supplementary table 3. The prevalence of number of domains dependency in IADL.**

| **Gender** | **Age groups** | **Without dependency (%)** | **Dependent One domain (%)** | **Dependent in**  **2-3 domains (%)** | **Dependent in**  **≥ 4 domains (%)** |
| --- | --- | --- | --- | --- | --- |
|  |  | **Point estimation**  **(95% CI)** | **Point estimation**  **(95% CI)** | **Point estimation**  **(95% CI)** | **Point estimation**  **(95% CI)** |
| **Females** | **60 – 64 years** | 41.53 (36.16 – 47.11) | 30.99 (26.30 – 36.10) | 22.36 (17.99 – 27.45) | 5.11 (3.15 – 8.19) |
|  | **65 – 69 years** | 33.65 (29.46 – 38.11) | 30.21 (26.44 – 34.27) | 32.69 (28.81 – 36.83) | 3.44 (2.18 – 5.39) |
|  | **70 – 74 years** | 25.84 (19.89 – 32.85) | 24.16 (18.32 – 31.14) | 35.95 (29.50 – 42.96) | 14.04 (9.60 – 20.09) |
|  | **75 – 79 years** | 15.91 (10.49 – 23.40) | 18.18 (12.41 – 25.84) | 40.15 (31.66 – 49.28) | 25.76 (18.99 – 33.94) |
|  | **80 – 84 years** | 5.80 (2.20 – 14.41) | 13.04 (6.97 – 23.09) | 47.83 (35.66 – 60.25) | 33.33 (23.17 – 45.32) |
|  | **≥ 85 years** | N.A. | 12.50 (5.38 – 26.43) | 22.50 (12.20 – 37.75) | 65.00 (48.26 – 78.71) |
|  | **All age groups *** | 28.84 (26.30 – 31.51) | 25.69 (23.40 – 28.13) | 31.17 (28.71 – 33.73) | 14.30 (12.40 – 16.44) |
|  | **All age groups **** | 28.50 (25.98 – 31.15) | 25.56 (23.27 – 27.98) | 31.64 (29.18 – 34.21) | 14.30 (12.40 – 16.45) |
| **Males** | **60 – 64 years** | 60.99 (55.05 – 66.63) | 26.24 (21.41 – 31.72) | 12.05 (8.75 – 16.38) | 0.71 (0.18 – 2.79) |
|  | **65 – 69 years** | 62.91 (58.12 – 67.46) | 23.71 (19.94 – 27.93) | 10.33 (7.73 – 13.67) | 3.05 (1.79 – 5.14) |
|  | **70 – 74 years** | 56.50 (50.04 – 62.75) | 24.50 (19.23 – 30.67) | 13.50 (9.43 – 18.95) | 5.50 (3.08 – 9.64) |
|  | **75 – 79 years** | 39.33 (32.04 – 47.13) | 24.67 (18.29 – 32.38) | 23.33 (17.27 – 30.73) | 12.67 (8.17 – 19.13) |
|  | **80 – 84 years** | 34.66 (24.93 – 45.88) | 28.00 (19.17 – 38.93) | 29.33 (20.17 – 40.54) | 8.00 (3.63 – 16.71) |
|  | **≥ 85 years** | 20.70 (9.76 – 38.11) | 17.24 (7.27 – 35.63) | 31.03 (17.09 – 49.56) | 31.03 (17.76 – 48.39) |
|  | **All age groups *** | 53.03 (50.02 – 56.01) | 24.81 (22.17 – 27.65) | 16.11 (13.93 – 18.56) | 14.30 (12.40 – 16.44) |
|  | **All age groups **** | 53.74 (50.83 – 56.62) | 24.75 (22.20 – 27.48) | 15.65 (13.58 – 17.97) | 5.86 (4.61 – 7.41) |
| **Total Participants** | **60 – 64 years** | 50.76 (46.71 – 54.79) | 28.74 (25.26 – 32.49) | 17.48 (14.65 – 20.72) | 3.02 (1.91 – 4.77) |
|  | **65 – 69 years** | 46.78 (43.50 – 50.09) | 27.29 (24.61 – 30.15) | 22.65 (20.09 – 25.44) | 3.27 (2.29 – 4.64) |
|  | **70 – 74 years** | 42.06 (37.46 – 46.80) | 24.34 (20.35 – 28.83) | 24.07 (20.13 – 28.51) | 9.52 (6.95 – 12.92) |
|  | **75 – 79 years** | 28.37 (23.28 – 34.08) | 21.63 (16.96 – 27.17) | 31.20 (25.96 – 36.98) | 18.79 (14.60 – 23.86) |
|  | **80 – 84 years** | 20.83 (14.74 – 28.07) | 20.83 (14.74 – 28.60) | 38.19 (30.26 – 46.81) | 20.14 (14.50 – 27.26) |
|  | **≥ 85 years** | 8.69 (7.98 – 17.91) | 14.49 (7.98 – 24.89) | 26.09 (17.29 – 37.32) | 50.72 (39.37 – 62.00) |
|  | **All participants*** | 40.80 (38.83 – 42.81) | 25.28 (23.50 – 27.15) | 23.65 (21.98 – 25.39) | 10.27 (9.09 – 11.57) |
|  | **All participants**** | 40.83 (38.87 – 42.81) | 25.32 (23.57 – 27.18) | 23.68 (22.07 – 25.38) | 10.02 (9.00 – 11.47) |

*Age and sex standardized based on the Iranian population in 2016

**Age-standardized based on World Health Organization Population 2000-2025

**Supplementary table 4. The prevalence of cognitive impairment, anorexia, and urinary and bowel incontinence in older adults in Bushehr city.**

| **Component** | **Age groups** | **Both sex (%)**  **Point estimation**  **(95% confidence interval)** | | | **Female (%)**  **Point estimation**  **(95% confidence interval)** | | | **Male (%)**  **Point estimation**  **(95% confidence interval)** | | |
| --- | --- | --- | --- | --- | --- | --- | --- | --- | --- | --- |
| **Cognitive impairment** | **60 – 64 years** | 44.48 | 40.80 | 48.22 | 52.23 | 46.99 | 57.42 | 35.92 | 30.61 | 41.59 |
|  | **65 – 69 years** | 47.37 | 44.04 | 50.72 | 58.02 | 53.51 | 62.39 | 34.27 | 29.95 | 38.87 |
|  | **70 – 74 years** | 55.29 | 50.45 | 60.03 | 67.42 | 60.71 | 73.47 | 44.50 | 37.78 | 51.42 |
|  | **75 – 79 years** | 68.09 | 62.30 | 73.36 | 74.24 | 66.11 | 80.98 | 62.67 | 54.71 | 69.99 |
|  | **80 – 84 years** | 70.55 | 62.43 | 77.55 | 72.46 | 60.82 | 81.69 | 68.83 | 57.63 | 78.19 |
|  | **≥ 85 years** | 73.53 | 62.09 | 82.49 | 75.00 | 59.72 | 85.85 | 72.41 | 53.27 | 85.80 |
|  | **All participants *** | 53.58 | 51.56 | 55.59 | 61.58 | 58.70 | 64.38 | 45.59 | 42.59 | 48.62 |
|  | **All participants **** | 53.74 | 51.70 | 55.77 | 62.02 | 59.17 | 64.78 | 44.94 | 42.02 | 47.89 |
| **Anorexia and malnutrition** | **60 – 64 years** | 1.51 | 0.78 | 2.89 | 2.23 | 1.06 | 4.62 | 0.71 | 0.18 | 2.80 |
|  | **65 – 69 years** | 0.84 | 0.42 | 1.68 | 1.34 | 0.63 | 2.80 | 0.23 | 0.03 | 1.65 |
|  | **70 – 74 years** | 2.12 | 1.08 | 4.11 | 2.81 | 1.19 | 6.50 | 1.50 | 0.49 | 4.53 |
|  | **75 – 79 years** | 1.06 | 0.35 | 3.22 | 1.52 | 0.39 | 5.69 | 0.67 | 0.09 | 4.62 |
|  | **80 – 84 years** | 1.37 | 0.34 | 5.33 | 2.90 | 0.72 | 10.89 | N.A. | N.A. | N.A. |
|  | **≥ 85 years** | 5.88 | 2.20 | 14.77 | 7.50 | 2.42 | 20.95 | 3.45 | 0.48 | 20.92 |
|  | **All participants *** | 1.62 | 1.11 | 2.35 | 2.39 | 160 | 3.54 | 0.84 | 0.39 | 1.80 |
|  | **All participants **** | 1.62 | 1.12 | 2.33 | 2.35 | 1.59 | 3.47 | 0.82 | 0.40 | 1.70 |
| **Urinary incontinence** | **60 – 64 years** | 21.11 | 17.85 | 24.77 | 31.31 | 26.24 | 36.87 | 9.86 | 6.94 | 13.82 |
|  | **65 – 69 years** | 26.13 | 23.41 | 29.05 | 34.23 | 30.35 | 38.32 | 16.20 | 12.92 | 20.12 |
|  | **70 – 74 years** | 28.31 | 23.86 | 33.22 | 36.52 | 29.93 | 43.65 | 21.00 | 15.79 | 27.37 |
|  | **75 – 79 years** | 34.75 | 29.78 | 40.08 | 44.70 | 36.73 | 52.95 | 26.00 | 20.09 | 32.94 |
|  | **80 – 84 years** | 38.36 | 30.71 | 46.63 | 44.93 | 34.10 | 56.25 | 32.47 | 22.95 | 43.70 |
|  | **≥ 85 years** | 33.82 | 22.93 | 46.75 | 45.00 | 29.87 | 61.11 | 17.86 | 7.58 | 36.56 |
|  | **All age groups *** | 27.08 | 25.36 | 28.87 | 36.28 | 33.60 | 39.05 | 17.59 | 15.51 | 19.86 |
|  | **All age groups **** | 27.40 | 25.68 | 29.20 | 36.44 | 33.79 | 39.18 | 17.65 | 15.64 | 19.86 |
| **Stool incontinence** | **60 – 64 years** | 0.67 | 0.25 | 1.77 | 0.64 | 0.16 | 2.52 | 0.50 | 0.18 | 2.77 |
|  | **65 – 69 years** | 0.32 | 0.10 | 0.98 | 0.38 | 0.10 | 1.52 | 0.24 | 0.03 | 1.66 |
|  | **70 – 74 years** | 0.79 | 0.26 | 2.43 | 1.12 | 0.28 | 4.41 | 0.50 | 0.07 | 3.45 |
|  | **75 – 79 years** | 1.77 | 0.74 | 4.17 | 1.52 | 0.38 | 5.91 | 1.13 | 0.66 | 5.94 |
|  | **80 – 84 years** | 1.37 | 0.34 | 5.29 | 1.45 | 0.20 | 9.69 | 1.29 | 0.18 | 8.71 |
|  | **≥ 85 years** | 4.41 | 1.42 | 12.90 | 5.00 | 1.24 | 18.12 | 3.51 | 0.50 | 21.53 |
|  | **All age groups *** | 0.97 | 0.48 | 1.94 | 1.07 | 0.58 | 1.98 | 0.97 | 0.48 | 1.94 |
|  | **All age groups **** | 0.91 | 0.46 | 1.78 | 1.07 | 0.58 | 1.95 | 0.91 | 0.46 | 1.78 |

*Age and sex standardized based on the Iranian population in 2016

**Age-standardized based on World Health Organization Population 2000-2025

**Supplementary table 5. The prevalence of depression and its severity in older adults in Bushehr city.**

| **Variables** | | **Without depression (%)** | | | **Mild Depression (%)** | | | **Moderate Depression (%)** | | | **Severe Depression (%)** | | |
| --- | --- | --- | --- | --- | --- | --- | --- | --- | --- | --- | --- | --- | --- |
| **Gender** | **Age groups** | **Point estimation (%)** | | | **Point estimation (%)** | | | **Point estimation (%)** | | | **Point estimation (%)** | | |
| **Total Participants** | **60 – 64 years** | 75.42 | 71.80 | 78.72 | 16.50 | 13.76 | 19.66 | 4.04 | 2.73 | 5.95 | 4.04 | 2.72 | 5.97 |
|  | **65 – 69 years** | 74.66 | 71.76 | 77.35 | 15.91 | 13.65 | 18.46 | 6.04 | 4.72 | 7.71 | 3.39 | 2.38 | 4.82 |
|  | **70 – 74 years** | 73.60 | 69.01 | 77.73 | 16.53 | 13.17 | 20.55 | 6.40 | 4.30 | 9.42 | 3.47 | 2.03 | 5.85 |
|  | **75 – 79 years** | 73.67 | 68.34 | 78.38 | 16.01 | 12.25 | 20.67 | 4.98 | 2.91 | 8.40 | 5.34 | 3.18 | 8.84 |
|  | **80 – 84 years** | 74.66 | 67.14 | 80.94 | 15.07 | 10.14 | 21.81 | 5.48 | 2.78 | 10.52 | 4.79 | 2.29 | 9.78 |
|  | **≥ 85 years** | 58.82 | 47.57 | 69.23 | 22.06 | 13.76 | 33.41 | 11.76 | 6.01 | 21.76 | 7.35 | 3.16 | 16.18 |
|  | **All participants *** | 73.88 | 72.01 | 75.67 | 16.47 | 14.98 | 18.07 | 5.48 | 4.60 | 6.52 | 4.17 | 3.40 | 5.10 |
|  | **All participants **** | 73.73 | 71.84 | 75.54 | 16.48 | 15.01 | 18.07 | 5.61 | 4.71 | 6.68 | 4.17 | 3.42 | 5.08 |
| **Female** | **60 – 64 years** | 63.14 | 57.66 | 68.30 | 24.36 | 19.95 | 29.38 | 5.77 | 3.69 | 8.92 | 6.73 | 4.43 | 10.10 |
|  | **65 – 69 years** | 64.67 | 60.67 | 68.48 | 21.81 | 18.44 | 25.61 | 8.11 | 6.07 | 10.75 | 5.41 | 3.73 | 7.76 |
|  | **70 – 74 years** | 58.52 | 50.97 | 65.70 | 25.00 | 19.25 | 31.79 | 10.80 | 7.04 | 16.20 | 5.68 | 3.08 | 10.23 |
|  | **75 – 79 years** | 60.31 | 51.90 | 68.14 | 24.43 | 17.95 | 32.32 | 6.87 | 3.71 | 12.38 | 8.40 | 4.72 | 14.50 |
|  | **80 – 84 years** | 57.97 | 46.83 | 68.35 | 24.64 | 16.15 | 35.70 | 8.70 | 3.98 | 17.94 | 8.70 | 3.92 | 18.17 |
|  | **≥ 85 years** | 45.00 | 30.99 | 59.85 | 25.00 | 13.86 | 40.84 | 20.00 | 10.35 | 35.12 | 10.00 | 3.88 | 23.42 |
|  | **All age groups *** | 60.96 | 57.93 | 63.91 | 23.92 | 21.43 | 26.60 | 8.33 | 6.81 | 10.16 | 6.78 | 5.43 | 8.44 |
|  | **All age groups **** | 60.95 | 57.95 | 63.87 | 23.91 | 21.43 | 26.58 | 8.40 | 6.88 | 10.20 | 6.74 | 5.41 | 8.37 |
| **Male** | **60 – 64 years** | 89.01 | 84.93 | 92.08 | 7.80 | 5.22 | 11.50 | 2.13 | 0.96 | 4.64 | 1.06 | 0.35 | 3.23 |
|  | **65 – 69 years** | 86.82 | 83.02 | 89.88 | 8.71 | 6.32 | 11.89 | 3.53 | 2.08 | 5.94 | 0.94 | 0.35 | 2.49 |
|  | **70 – 74 years** | 86.93 | 81.74 | 90.82 | 9.05 | 5.78 | 13.87 | 2.51 | 1.06 | 5.85 | 1.51 | 0.49 | 4.52 |
|  | **75 – 79 years** | 85.33 | 78.64 | 90.19 | 8.67 | 5.04 | 14.50 | 3.33 | 1.40 | 7.75 | 2.67 | 0.82 | 8.30 |
|  | **80 – 84 years** | 89.61 | 80.50 | 94.74 | 6.49 | 2.72 | 14.73 | 2.60 | 0.65 | 9.82 | 1.30 | 0.18 | 8.68 |
|  | **≥ 85 years** | 78.57 | 60.45 | 89.79 | 17.86 | 7.84 | 35.70 | N.A. | N.A. | N.A. | 3.57 | 0.50 | 21.51 |
|  | **All age groups *** | 87.14 | 84.89 | 89.11 | 8.81 | 7.18 | 10.78 | 2.56 | 1.78 | 3.66 | 1.48 | 0.86 | 2.55 |
|  | **All age groups **** | 87.11 | 84.94 | 89.00 | 8.80 | 7.23 | 10.68 | 2.62 | 1.84 | 3.72 | 1.47 | 0.87 | 2.48 |

*Age and sex standardized based on Iranian population in 2016

**Age standardized based on World Health Organization Population 2000-2025
